# Synergistic Biostimulatory Action: Designing the Next Generation of Plant Biostimulants for Sustainable Agriculture

**DOI:** 10.3389/fpls.2018.01655

**Published:** 2018-11-13

**Authors:** Youssef Rouphael, Giuseppe Colla

**Affiliations:** ^1^Department of Agricultural Sciences, University of Naples Federico II, Portici, Italy; ^2^Department of Agriculture and Forest Sciences, University of Tuscia, Viterbo, Italy

**Keywords:** biostimulant 2.0, humic acids, microbial inoculants, microbiome, protein hydrolysate, physiological mechanisms, seaweed extracts, synergistic properties

## Abstract

Over the past 10 years, interest in plant biostimulants (PBs) has been on the rise compelled by the growing interest of scientists, extension specialists, private industry, and growers in integrating these products in the array of environmentally friendly tools that secure improved crop performance and yield stability. Based on the new EU regulation PBs are defined through claimed agronomic effects, such as improvement of nutrient use efficiency, tolerance to abiotic stressors and crop quality. This definition entails diverse organic and inorganic substances and/or microorganisms such as humic acids, protein hydrolysates, seaweed extracts, mycorrhizal fungi, and N-fixing bacteria. The current mini-review provides an overview of the direct (stimulatory on C and N metabolism) and indirect (enhancing nutrient uptake and modulating root morphology) mechanisms by which microbial and non-microbial PBs improve nutrient efficiency, plant performance, and physiological status, resilience to environmental stressors and stimulate plant microbiomes. The scientific advances underlying synergistic and additive effects of microbial and non-microbial PBs are compiled and discussed for the first time. The review identifies several perspectives for future research between the scientific community and private industry to design and develop a second generation of PBs products (biostimulant 2.0) with specific biostimulatory action to render agriculture more sustainable and resilient.

## Defining Plant Biostimulants: Action, Composition, Regulations

According to the United Nations estimates, the global population will expand from approximately 7.5 billion presently to more than 9.7 billion by 2050, compelling modern agriculture to become increasingly more efficient by producing more food in an eco-friendly and sustainable way. An innovative technology addressing these important challenges involves the development of novel plant biostimulants (PBs) and effective methods for their application (Calvo et al., 2014; Bulgari et al., 2015; Yakhin et al., 2017). The global biostimulant market is estimated today at about $ 2.0 billion, expected to reach $ 3.0 billion by 2021 at an annual growth rate of 10–12% (Dunhamtrimmer.com, 2018). Based on the Marketsandmarkets.com (2017) database, Europe is the largest PBs market accounting for 34% of the worldwide market share, followed by the North American and Asian-Pacific biostimulant markets which account roughly for 23 and 22%, respectively, of the global market. The main factors driving the rapid growth of the biostimulants market have been associated with: (i) the growing availability of novel biostimulant products addressing specific agronomic needs; (ii) the need to promote a more efficient and effective use of synthetic chemicals and mineral fertilizers; (iii) the increasing frequency of adverse environmental conditions for crop growth and productivity.

Plant biostimulants are natural compounds that trigger physiological and molecular processes modulating crop yield and quality, though their primary function is neither to supply nutrients (fertilizers) nor to protect plants against soilborne or foliar pests and pathogens (Plant Protection Products) (du Jardin, 2015). Therefore, PBs were initially defined by *what they are not*. The term “biostimulant” was first proposed by Zhang and Schmidt (1997) to denote “*materials that, in minute quantities, promote plant growth*.” The PBs referred to in the former article were humic substances and macro-algal extracts whose stimulatory effect on plants was essentially suggesting a hormonal mode of action.

The term was subsequently adopted by many researchers, regulators, and extension specialists to denote “Substance(s) and/or micro-organisms whose function is to stimulate natural processes that enhance nutrient uptake, nutrient use efficiency, tolerance to abiotic stress, and crop quality” [European Biostimulant Industry Council (EBIC)] ^1^. From a regulatory point of view, there is no agreement globally over the definition of PBs and many EU and non-EU countries lack a specific legal framework (Yakhin et al., 2017; Caradonia et al., 2018; Rouphael et al., 2018). Recently the EU decided to reshape the existing Fertilizers Regulations to promote internal market operations for fertilizing products and also to establish a common legal framework for PBs, currently fragmented across Member States (Caradonia et al., 2018; Rouphael et al., 2018). Under the new regulation, “plant biostimulants will be CE marked as fertilizing products stimulating plant nutrition processes independently of the products’ nutrient content with the sole aim of improving one or more of the following characteristics of the plant **and the plant rhizosphere or phyllosphere**: nutrient use efficiency (NUE), tolerance to abiotic stress, crop quality, **availability of confined nutrients in the soil and rhizosphere, humification and degradation of organic compounds in the soil**” (In **bold**: amendments adopted by the European Parliament on October 24, 2017, still to be discussed with the European Council and the Commission; European Commission, 2016). PBs are thus to be defined on the basis of claimed effects, in other words “by the plant response they elicit rather than by their makeup,” since the category entails diverse organic and inorganic substances and/or microorganisms such as humic acids, protein hydrolysates, seaweed extracts, mycorrhizal fungi, and N-fixing bacteria (du Jardin, 2015; Rouphael et al., 2018).

Significant advancements have been made in studying and unraveling the mode of action(s) of PBs, thanks to the omics science, in particular genomic and transcriptomic tools, as well as through high-throughput phenotyping technologies (Calvo et al., 2014; Lucini et al., 2015; Povero et al., 2016; Bulgari et al., 2017; Rouphael et al., 2018). In addition, over the past 10 years, private industries have been investing in increasing the effectiveness of their formulations through blends of microbial and non-microbial PBs. However, the approach used for the development of these mixtures is mainly empirical without solid scientific evidence of interactive effects (i.e., antagonism, additive, or synergistic) between their microbial and non-microbial components. This approach rests on the premise that the more biostimulants are combined in a mixture the better that will work; however, *more isn’t always better*. The identification of synergistic properties among PBs based on reasonable scientific hypotheses and sound experimental approaches (testing PBs alone and in combination), rather than a *try-and-see approach*, can be pivotal for developing novel and target-specific biostimulant products able to increase NUE and improve plant resilience to environmental stressors.

Taking this background into consideration, limited published data is available concerning the interaction between microbial and non-microbial PBs. The current mini-review article examines the mode of actions/mechanisms by which microbial and non-microbial PBs affect nutrient uptake efficiency, plant performance, and tolerance for abiotic stressors. Subsequently, the scientific advances addressing the synergistic and additive effects among microbial and non-microbial PBs are reviewed and discussed. Finally, the current mini-review identifies the challenges ahead and the future direction of research to develop and exploit a second generation of effective biostimulants rendering agriculture more sustainable and resilient.

## Mechanisms Implicated in Plant Biostimulatory Effects on Crop Physiology and Agronomic Performance

### Organic Non-microbial Plant Biostimulants

Based on the latest draft of the European Commission (2016), organic non-microbial PBs include natural substances such as humic acids (HA), protein hydrolysates (PH), and seaweed extracts (SWE), with the first two categories commanding half of the market share, whereas the SWE segment amounts to 37% of the total market.

Humic substances such as humic and fulvic acids are natural organic molecules originating from the biological and chemical transformations of dead organic matter (Nardi et al., 2007; Canellas et al., 2015). Humic substances are generally applied as soil drench and in some cases (fulvic acids) through foliar application (Halpern et al., 2015). Humic substances have been perceived for long as primordial components of soil fertility and structure, acting on chemical, physical as well as biological properties of soils (du Jardin, 2015). The biostimulation action of HAs on soil nutrient availability and uptake has been attributed to several mechanisms affecting soil processes and plant physiology including: (i) improving soil structure, (ii) increasing cation exchange capacity and neutralizing soil pH, (iii) improving solubility of phosphorus by interfering with Ca-phosphate precipitation and also by increasing the availability of micronutrients by preventing leaching, (iv) improving lateral root induction and hair growth due to the auxin-like activity, which triggers plasma membrane H^+^-ATPase activity, and (v) stimulating nitrate assimilation through the upregulation of the target enzymes (NR, GDH, and GER) involved in this process (Pinton et al., 1999; Delgado et al., 2002; García-Mina et al., 2004; Schmidt et al., 2007; Halpern et al., 2015; Zandonadi et al., 2016; De Pascale et al., 2017). The biostimulatory action of HAs is highly influenced by soil fertility conditions, HAs being more effective under soil conditions of poor fertility and low organic matter content (du Jardin, 2015). Variability in the effects of HAs is also due to the source of humic substances, with higher plant performance obtained in response to HAs extracted from humidified organic matter (e.g., peat), composts and vermicomposts rather than those coming from fossil humus (du Jardin, 2015).

In addition to the indirect and direct effects of HAs on plant metabolism and physiology, several studies demonstrated their biostimulatory activity in terms of stress protection particularly against salinity and drought (Türkmen et al., 2004; Paksoy et al., 2010; Aydin et al., 2012; García et al., 2012; Petrozza et al., 2014). Presumed mechanisms involved in salt and drought tolerance are: (i) reducing hydrogen peroxide and lipid peroxidation, (ii) increasing proline content, (iii) differential regulation of gene expression, and (iv) improving root growth as well as the chemical, microbiological and physical properties of soil (Calvo et al., 2014; Battacharyya et al., 2015).

According to Colla et al. (2015a, 2017b) animal- as well as plant-based PH represent an important category of organic non-microbial PBs, having as main components a mixture of free amino acids, oligo- and polypeptides which act as *signaling molecules*. PHs are mainly applied as foliar spray and to a lesser extent as a substrate drench and as seed treatment (Colla et al., 2015a). In several greenhouse and open-field studies, PHs demonstrated an important role as PBs by triggering physiological and molecular processes that stimulate growth and productivity thus mitigating the impact of several abiotic stressors on crops (Colla et al., 2017b). Direct effects behind the biostimulation activity and abiotic stress tolerance of PHs include: (i) triggering of key enzymes involved in N assimilation (NR, NiR, GS, and GOCAT) and C metabolism (citrate synthase, malate, and isocitrate dehydrogenase), (ii) heightened auxin- and gibberellin-like activities, and (iii) increase in antioxidant enzyme activity, pigment biosynthesis, and production of secondary metabolites (Schiavon et al., 2008; Ertani et al., 2009, 2013, 2017; Rouphael et al., 2017a, 2018; Sestili et al., 2018). In addition to the direct effect of PHs, indirect effects on crop performance and nutritional status have been also demonstrated when PHs were applied as foliar spray or as substrate drench (Colla et al., 2017b). In fact, the application of PHs has enhanced nutrient uptake by increasing the effective volume of soil exploited by the root system, through their effects on root system architecture, in particular the increase in root hair diameter, density and length (Colla et al., 2014, Colla et al., 2017b). Moreover, in a recent review Colla et al. (2017b) reported that PHs can also affect plant microbiomes residing in both rhizosphere and phyllosphere, thus improving plant performance by altering development and physiological processes, resulting in higher water and nutrient uptake as well as enhanced resilience against major environmental threats.

Seaweeds are brown, green, and red macroalgae, available on the biostimulant market as powder, granular form and as liquid extracts and may be applied as foliar sprays or side-dressed near the root. The major components of commercial SWE are polysaccharides, followed by phenolics, vitamins precursors, osmolytes (mannitol), phytohormones, and hormone-like compounds (Battacharyya et al., 2015). Brown macroalgae, with *Ascophyllum*, *Ecklonia*, *Fucus*, *Laminaria*, and *Sargassum* as main genera, are widely used in crops as PBs for their plant-growth promoting benefits, abiotic stress resistance, and improved postharvest quality and shelf-life (Vernieri et al., 2006; Khan et al., 2009; Craigie, 2011; Rouphael et al., 2017b). The beneficial effects of SWE may be attributed to several growth enhancing mechanisms like (i) physiological (delayed senescence) and biochemical changes (increased micronutrients), (ii) improved WUE (improved stomatal conductance and root-to-shoot ratio), (iii) differential regulation of genes (*CBF3*, *SOS*, *RD22*), and (iv) rhizosphere effects (increased activity of rhizobacteria and mycorrhizae) (Battacharyya et al., 2015). Although significant advancements have been shedding light on the modes of action/mechanisms of the organic non-microbial PBs, additional research is needed to optimize the use of PBs including the standardization of their raw materials, characteristics, extraction methods as well as identifying the optimal application time, dose and mode for each species and set of environmental conditions.

### Microbial Plant Biostimulants

The use of microbial-based biostimulants such as plant growth promoting rhizobacteria (PGPR) of strains belonging to the genera *Azospirillum*, *Azotobacter*, and *Rhizobium* spp. as well as mycorrhizal fungi are highly considered as promising means not only to secure yield stability under low-input conditions (i.e., N and/or P deficiency) but also as a tool to solving some environmental constraints (Rouphael et al., 2015; Ruzzi and Aroca, 2015). In fact, several studies (Lace et al., 2015; Ruzzi and Aroca, 2015; Fiorentino et al., 2018) demonstrated that PGPR and endophytic fungi including mycorrhizal fungi can modulate quantitatively and qualitatively the rhizosphere microbial population with positive impact on the soil ecosystem.

The phytostimulation effect of PGPR and mycorrhizal fungi under both optimal and suboptimal conditions could be attributed to several direct and indirect mechanisms including: (i) improved uptake and translocation of nutrients including N and P and micronutrients (Fe, Zn, and Mn), (ii) more vigorous root system apparatus (higher root biomass, surface area, and number of lateral roots) especially in crops having a taproot system (e.g., carrot) or a shallow root apparatus (e.g., onion), (iii) improved water relations and photosynthetic capacity, (iv) stronger antioxidative defense system, (v) regulation of plant hormones (auxins, ABA, cytokinins, ethylene, and gibberellins), (vi) promotion of nutrient transporters (NRT1.1, NRT2, NAR2.2, AMT, Pht1, and PT2-1) activity, and (vii) production of enzymes (phosphatases) and/or excretion of low- (amino acids, sugars, organic acids, and phenolics) and high-molecular weight organic compounds (mucilage and proteins) in the rhizosphere (Hayat et al., 2010; Candido et al., 2013, 2015; Colla et al., 2015a,b; Rouphael et al., 2015; Saia et al., 2015; De Pascale et al., 2017; Bitterlich et al., 2018).

## Exploiting Synergistic Interactions Among Plant Biostimulants: Moving Toward the Next Generation of Biostimulants

The agricultural sector’s pursuit of decreased reliance on organic and inorganic fertilizers by improving NUE and mitigating the negative impact of environmental stress factors and soil degradation (biological, chemical, and physical) on crop growth and productivity. Biostimulants have the capacity to improve NUE and reduce abiotic stress on crops, and these are claims supporting their placing on the market. In fact, Colla et al. (2017a) demonstrated that under the same fertilization program (rates and time of application) the application of PBs improved the NUE and thus the yield of greenhouse fresh tomato by 6.6–11.0%. Similarly, an endophytic fungal consortium inoculum boosted the marketable yield of open-field zucchini squash and lettuce by 14 and 70%, respectively, compared to non-inoculated plants under the same fertilization regime (Colla et al., 2015b). Thus, application of microbial and non-microbial PBs could be considered an efficient approach to boost yield without raising the rate of applied nutrients (i.e., higher NUE). Crops are also faced with multiple/combined abiotic constraints, particularly drought, salinity, and heat. These are the ones forecasted to escalate most according to climate change models and challenge yield stability (Mittler, 2006; Suzuki et al., 2014). Thus, research on the potential synergistic effects among PBs should be at the core of future efforts in addressing global food security, complemented by sustainable and optimized use of nutrients.

In terms of efficacy, there are three types of interactions implicating microbial and/or non-microbial PBs: they can be antagonistic, additive or synergistic based on their effective action. In antagonistic interactions the overall effect of the PBs applied is less than the additive effect of the PBs applied independently. This type of interaction is normally associated to the antagonistic non-target action of several *Trichoderma* spp. through mycoparasitism on the mycorrhizal fungi mycelium (McAllister et al., 1994; Martinez et al., 2004; De Jaeger et al., 2010). For instance, De Jaeger et al. (2011) reported a sharp decrease in P uptake in mycorrhized plants because of the disruption of the hyphae continuity of *Rhizophagus intraradices* by *T. harzianum*. In the case of additive interaction, the applied PBs have a similar type of effect on the plants, hence their combined effect equals the sum of their independent effects. Finally, synergistic interaction is observed when the combined effect of the applied PBs exceeds their additive effects when applied independently under the same conditions.

**FIGURE 1:**
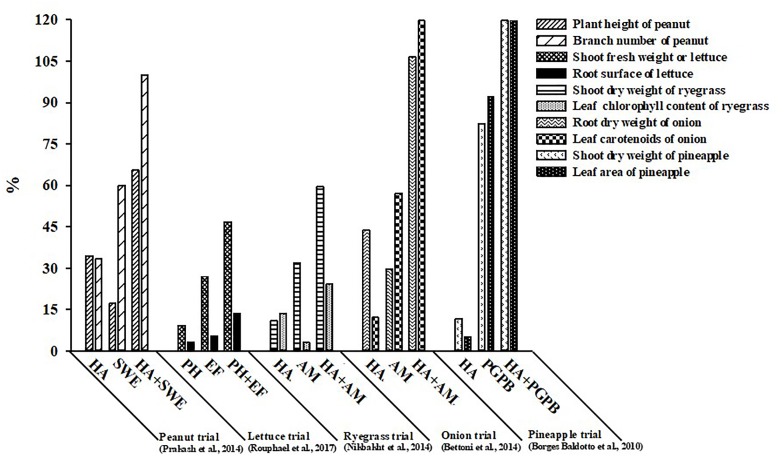
The relative effect of the various categories of non-microbial and microbial plant biostimulants, separately or in synergistic combination, on morphological and biochemical traits of open-field and protected cultivation crops (peanut, lettuce, perennial ryegrass, onion, and pineapple).

In recent years, limited experimental studies testing the additive and/or synergistic effects of various PBs categories, have demonstrated that combinations of non-microbial PBs or microbial inoculants with HA, SWE, or PH give more reproducible benefits to plant growth and production (Borges Baldotto et al., 2010; Bettoni et al., 2014; Nikbakht et al., 2014; Prakash et al., 2014; Rouphael et al., 2017a). For instance, groundnut sprayed at biweekly intervals with HA or SWE increased plant height and branching by 34.5 and 33% (for HA) and 17.2 and 60.0% (for SWE), respectively, in comparison to the untreated control treatment, whereas the applications of both PBs together (SWE:HA) exhibited a synergistic interaction with higher increase (65.0 and 100%, respectively) compared to the sum of the independent biostimulant effects (Figure 1; Prakash et al., 2014). In the same study, improvement in plant growth was associated to the stimulation of N uptake and chlorophyll biosynthesis which may have improved the photosynthetic activity, triggering the translocation of photosynthates to the sinks. The application of HA 1 day after transferring seedlings or the application of liquid mycorrhizal inoculum (*R. intraradices*) to the roots 2 days after transplanting resulted in a significant increase in onion root dry weight and leaf carotenoids by 43.9 and 12.1% (for HA) and by 29.6 and 57.1% (for mycorrhizal fungi), respectively, whereas the application of both HA and mycorrhizal fungi induced a synergistic effect with the highest accumulation of the two parameters measured (106.7 and 123.6%) (Figure 1; Bettoni et al., 2014). The presumed mode of action involved in the stimulation of crop performance was linked to enhanced nutrient availability driven by the synergistic action of HA and mycorrhizal fungi applied in combination. Similarly, in perennial ryegrass the combination of pre-sowing the substrate with mycorrhizal fungi (*R. intraradices*) and HA spray applications at 30-day intervals was more effective in enhancing root biomass and chlorophyll biosynthesis than either application alone (Figure 1; Nikbakht et al., 2014). Dipping the roots of micropropagated pineapple plantlets for 24 h before planting in a suspension of vermicompost derived-HA followed by application of HA to the basal leaf axils of plants at 14-day intervals and/or dipping of the roots before planting in a PGPB cell suspension for 30 min, increased the shoot dry weight and the leaf area of pineapple during the vitro acclimatization stage when applied separately but more so when applied in synergistic combination (Figure 1). Finally, Rouphael et al. (2017a) reported that the combination of an endophytic fungal consortium (*R. irregulare* BEG72 and *T. atroviride* MUCL45632) with weekly substrate drench applications of a plant-derived PH was more effective than microbial or non-microbial biostimulant applications alone in improving crop productivity (Figure 1). The beneficial effects of the combined biostimulants were associated with increased chlorophyll biosynthesis, the capability of maintaining higher photochemical activity in PSII, and also with a superior nutritional status of the leaf tissues.

## Conclusion and Challenges Ahead

The use of PBs in agriculture has greatly increased in the last 10 years, mostly due to their *multifaceted properties*. Significant advancements have been made in studying the physiological and biochemical mechanisms of PBs owing to the “omics” sciences and recently to the high-throughput phenotyping technologies. Nonetheless, additional research is required for confronting a number of open questions, such as: (1) which molecular mechanisms underlie the observed biostimulatory action? (2) what is the optimal method, time, rate of application and phenological stage for improving plant performance and resilience to stress and to what extend the plant species/cultivar, environment and management practices applied may affect these effects? (3) how effectively can the PBs modulate the microbial population quantitatively and qualitatively when applied as foliar spray, substrate drench or seed treatment? (4) how long do the PBs effects persist subsequently to their foliar application and how do factors such as leaf cuticle morphology and stomatal aperture interact with the different components of PBs and the target species in impacting leaf permeability and thus the efficacy of the product? and (5) what are the physiological and molecular mechanisms behind the synergistic properties among PBs and how can they be accounted for in developing novel and specific biostimulant products? While presently there seem to be more questions than answers, the findings of the few researchers that have attempted to unravel the complex biostimulation action behind PBs, particularly the synergistic properties, suggest that additional investment in research interaction between the scientific community and the private industry is required in order to develop a second generation of PBs products (biostimulant 2.0) with specific biostimulation action.

## Author Contributions

YR and GC had the original idea of Synergistic Biostimulatory Action and were both involved in writing the article.

## Conflict of Interest Statement

The authors declare that the research was conducted in the absence of any commercial or financial relationships that could be construed as a potential conflict of interest.

## Footnotes

1 www.biostimulants.eu
